# A Gas Chromatography-Mass Spectrometry Method to Determine Tramadol Abuse Using Urine Samples

**DOI:** 10.7759/cureus.75424

**Published:** 2024-12-09

**Authors:** Priyamvada Sharma, Vijayashree Rao, Lekhansh Shukla, Pratima Murthy

**Affiliations:** 1 Department of Clinical Psychopharmacology and Neurotoxicology, National Institute of Mental Health and Neurosciences, Bangalore, IND; 2 Centre for Addiction Medicine, Department of Psychiatry, National Institute of Mental Health and Neurosciences, Bangalore, IND; 3 Department of Psychiatry, National Institute of Mental Health and Neurosciences, Bangalore, IND

**Keywords:** abuse, gas chromatograph-mass spectrometry, solid-phase extraction, substance abuse, tramadol

## Abstract

Background

The synthetic opioid tramadol is widely used as a pain reliever. Unlike other opioids, it is used freely worldwide, unaffected by international controls resulting in abuse and accidental intoxication. Analytical methods are necessary to prove tramadol abuse because 30% of the drug is excreted unchanged.

Methodology

This study describes a sensitive, precise, and accurate gas chromatograph-mass spectrometry (GC-MS) method for tramadol quantification in biological samples using solid-phase extraction (SPE) for sample preparation.

Results

A total of 747 samples were analyzed for suspected tramadol abuse; 15% of samples were above cut-off with a mean of 341.0 ± 215 ng/mL. No interference from other substances was detected. Calibration was linear over the concentration range of 50-1,000 ng/mL with a correlation coefficient of >0.998. Recovery was 92.5% and precision was ≤5% (range = 2.68-5.58%).

Conclusions

The effectiveness of the SPE in assessing tramadol abuse was assessed with GC-MS. With good recovery, quick analysis times, simplicity, little matrix effect, and effectiveness, this method is regarded as novel and is capable of identifying abuse.

## Introduction

The synthetic opioid analgesic tramadol, also known as a serotonin-norepinephrine reuptake inhibitor (SNRI) and a centrally acting opioid, shares structural similarities with morphine and codeine. Tramadol is typically viewed as a lower-risk opioid option for the treatment of moderate-to-severe pain because of its excellent tolerability profile and multimodal mechanism of action [1]. Tramadol differs from other opioids and affects monoamines by changing pain-modulating neurotransmitters such as serotonin and norepinephrine [2]. Tramadol’s effects on serotonin and norepinephrine are comparable to those of other SNRIs such as duloxetine and venlafaxine [3-5]. There are 20 million opioid users in India, with 2.5 million using pharma opioids [6-8]. Many Southeast Asian countries are unable to regulate tramadol’s manufacture and usage as it is not listed on the international drug schedule [7,8]. In humans, tramadol is metabolized by the enzyme cytochrome P4502D6 (CYP2D6) and eliminated in the urine after conjugation with glucuronic acid and sulfate [9]. Urine screening is thought to be a reliable and specific method to detect its abuse. The Substance Abuse and Mental Health Services Administration [10] recommends a two-step testing procedure, which comprises an initial screening (lateral flow immunoassay) and confirmation using advanced techniques [11,12]. Several methods have been reported for the determination of tramadol, including gas chromatography-mass spectrometry (GC-MS), using plasma, saliva, hair, and biological materials [13,14]. However, most of them do not address the effect of multi-residual compounds coeluting in liquid-liquid extraction (LLE) or protein precipitation [15-17]. These co-extracted matrix components often increase the response, resulting in an inaccurate quantitation for the determination of tramadol in urine. Therefore, our study describes a rapid and sensitive method for the accurate determination of tramadol using GC-MS.

## Materials and methods

All chemicals used were of analytical grade. Bulk solvents and routine chemicals were procured from the SISCO research laboratory (Mumbai, India). Tramadol standard and internal standard (IS) were procured from Cerilliant Corporation, Texas, USA. The study was conducted from June 2017 to December 2021. Inverse binomial sampling was used. Samples were obtained from inpatient and outpatient departments of a tertiary deaddiction facility based in the southern part of India. Urine drug testing was offered as a routine clinical care service to the subjects undergoing treatment for which permission from the institutional ethics committee was obtained. Sociodemographic details including age, sex, marital status, educational status, family income, and occupational status were obtained for each subject. A total of 747 subjects with a history of tramadol use participated in the clinical interview.

Urine sample screening

In this study, 2-5 mL of urine was collected in labeled leak-proof sterile plastic containers. Initial screening was for adulteration (creatinine, specific gravity, nitrite, glutaraldehyde, pH, oxidant/pyridinium, chlorochromate) using adulteration strips from Abon, Inc (Northvale, NJ, USA). Unadulterated samples were subjected to multidrug (cannabis, morphine, benzodiazepines, cocaine, and amphetamine) and tramadol screening using commercial test cassettes from Alfa Scientific (Poway, CA, USA).

Preparation of stocks

Standard solutions (cis-tramadol HCL and IS tramadol-13C D3) were prepared in methanol, diluted to 10 g/mL, and kept at 20°C. Negative controls included drug-free human urine samples collected from healthy volunteers, held at 3°C for two weeks, and discarded.

Solid-phase extraction

To 500 µL of urine, 100 µL of 0.1 M phosphate buffer (pH 6) and 10 µL (1.0 µg/mL) tramadol IS was added. Solid-phase extraction (SPE) was done using a vacuum manifold procured from Agilent. The pretreated sample was loaded on preconditioned (with methanol and water) Bond-Elute C-18 cartridges (3 mL), followed by washing with 0.1 M acetic acid and methanol. Elution was done with ethyl acetate:acetonitrile:ammonia (8:2:0.2 v/v), and the sample was vacuum dried using a Genevac Vacuum evaporator. Before injection to GC-MS, derivatization was done with 50 µL of BSTFA (N, O-Bis (trimethylsilyl) trifluoroacetamide with 1% tri methylchlorosilane) at 80°C.

Confirmation and quantification by GC-MSD

**Figure 1:**
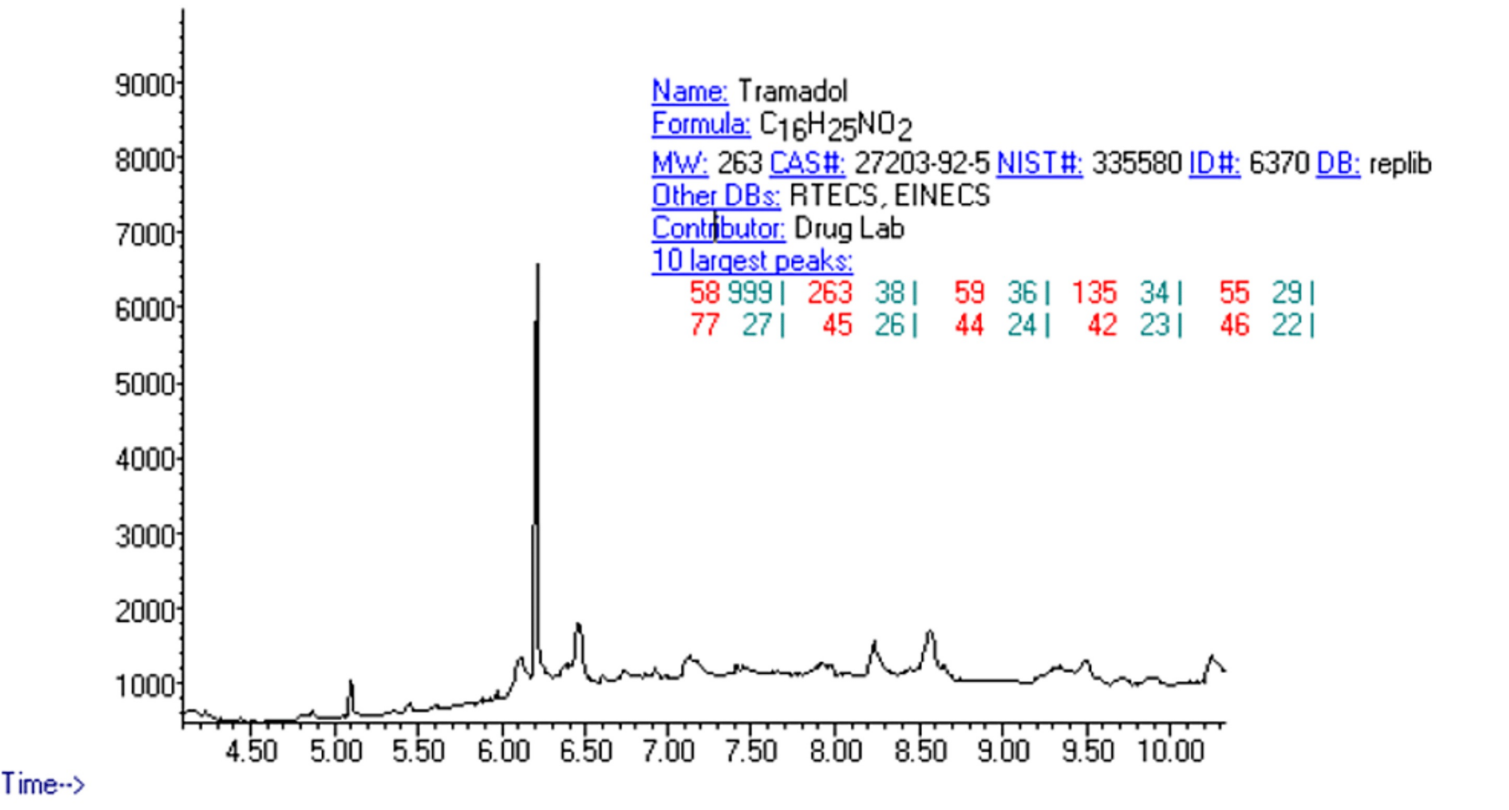
Tramadol chromatogram and masses

Agilent 7890-A GC coupled with 5975C MSD was used for sample analysis. Chromatographic separation was achieved on DB-5MS fused-silica capillary column and oven temperature was kept at 60°C for two minutes before gradually increasing to 200°C at a rate of 10°C/minute and finally to 250°C at a rate of 15°C/minute. At 250°C, the temperature was held for one minute. The inlet and transfer line temperatures were 250°C and 280°C, and the mass range selected was 50-550 amu, in electron ionization (EI) mode at 70 eV. Data were collected in the Selected-Ion Monitoring mode (SIM mode) and mass m/z qualifiers of 58 (target ion), 73, and 335 for tramadol. The Chemstation software was used (Figure 1).

Statistical analysis

The SPSS version 20.0 for Windows (IBM Corp., Armonk, NY, USA) was used to compute and analyze the results.

## Results

Method validation

Tramadol can be measured in urine samples using the current analytical technique. The potential of the technique is discussed below.

Linearity

**Figure 2:**
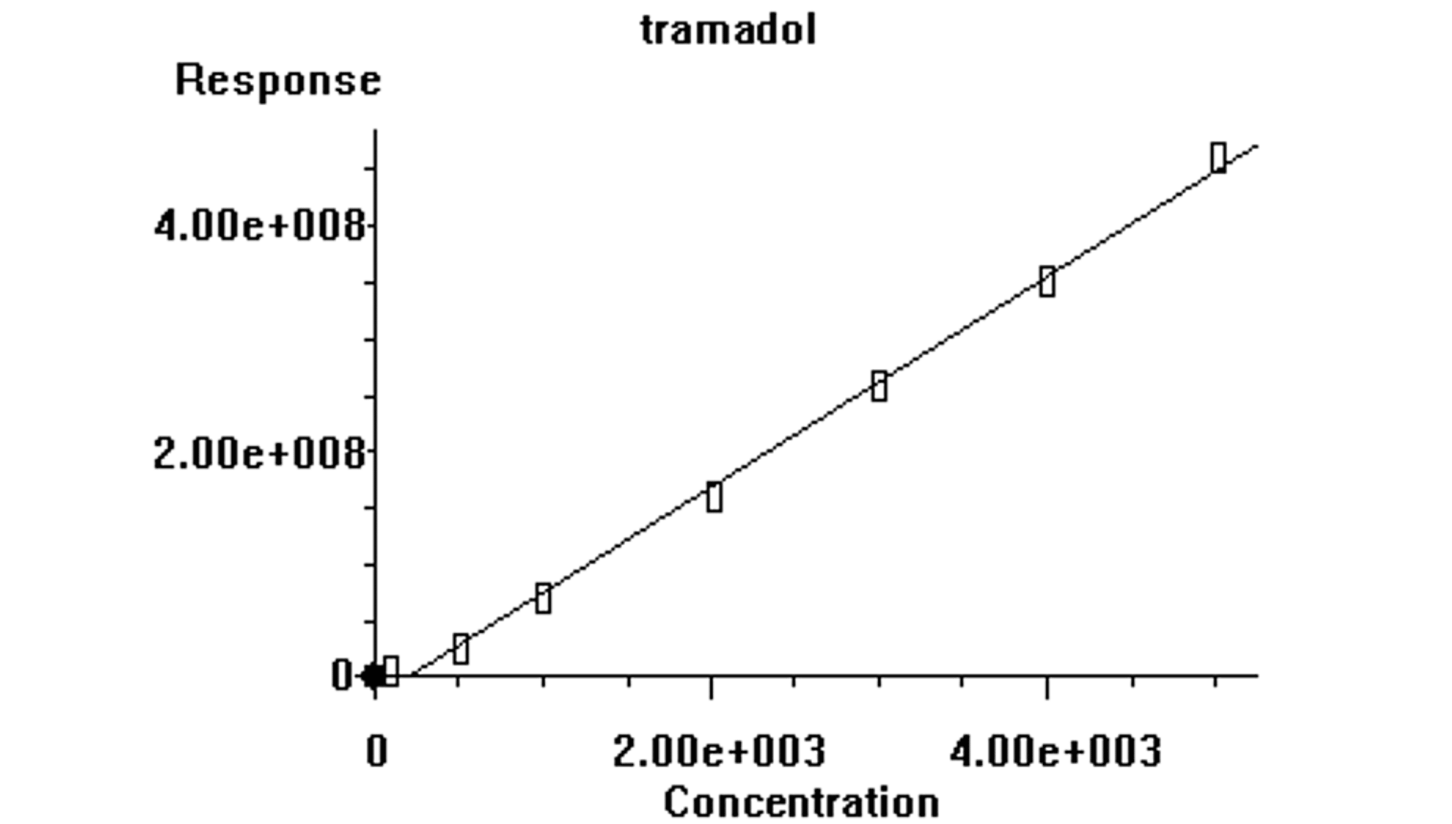
The calibration was done using the area of an analyte concentration versus the area of internal standard.

The calibration curve was linear over the concentration range of 50-1,000 ng/mL, and the correlation coefficient was ≥0.998 (Figure 2).

Sensitivity

The limit of detection (LOD) for tramadol was 20 ng/mL. The limit of quantification, i.e., the lowest concentration of analyte in a sample that can be reliably measured, was 50 ng/mL.

Precision and recovery

The precision was determined by repeatability (intraday) and intermediate precision (interday). Repeatability was evaluated by analyzing spiked blank urine six times per day at low (30 ng/mL), medium (500 ng/mL), and high (750 ng/mL) concentrations. The intermediate precision was evaluated by analyzing the same urine samples once daily for three days. The method offered excellent precision (interday: 4.6%, intraday: 4.4%) The mean recovery was ≥92.5% in urine for tramadol.

Matrix effect

The matrix effect was investigated using three lots of blanks from individual donors. The relative matrix effect of tramadol at three different concentrations of low (30 ng/mL), medium (500 ng/mL), and high (750 ng/mL) was ≤1.2%.

Sample stability

Ten independent urine samples were investigated for analyte stability, and the samples were found to be stable at room temperature for 24-48 hours. Three aliquots of low (30 ng/mL), medium (500 ng/mL), and high (750 ng/mL) concentrations were tested for short-term stability through freeze-thaw cycles (n = 3). Frozen samples were thawed naturally at 20°C/room temperature before analysis and recovery (Table 1).

**Table 1:** Stability of tramadol sample (biological) under various storage conditions.

Tramadol storage conditions	Spiked concentration (ng/mL)	Calculated concentration (ng/mL)	% Relative standard deviation
Storage at room temperature for eight hours	150	148	2.2
	750	742	4.4
Storage at -20°C for one week	150	149	3.1
	750	745	4.8
Freeze-thaw cycle (n = 3)	150	151	3.2
	750	148	5.2

Applicability of the method

The applicability of the method was demonstrated in the clinical samples. We analyzed 747 samples from June 2017 to December 2021 for tramadol use, of which 564 samples had values ≤100 ng/mL while 183 above cut-off values ranged from 100 ng/mL to 22,149.0 ng/mL. The mean was 176,611 ± 29,180.25 ng/ml. The samples with tramadol above calibration were processed with the required dilution.

**Figure 3:**
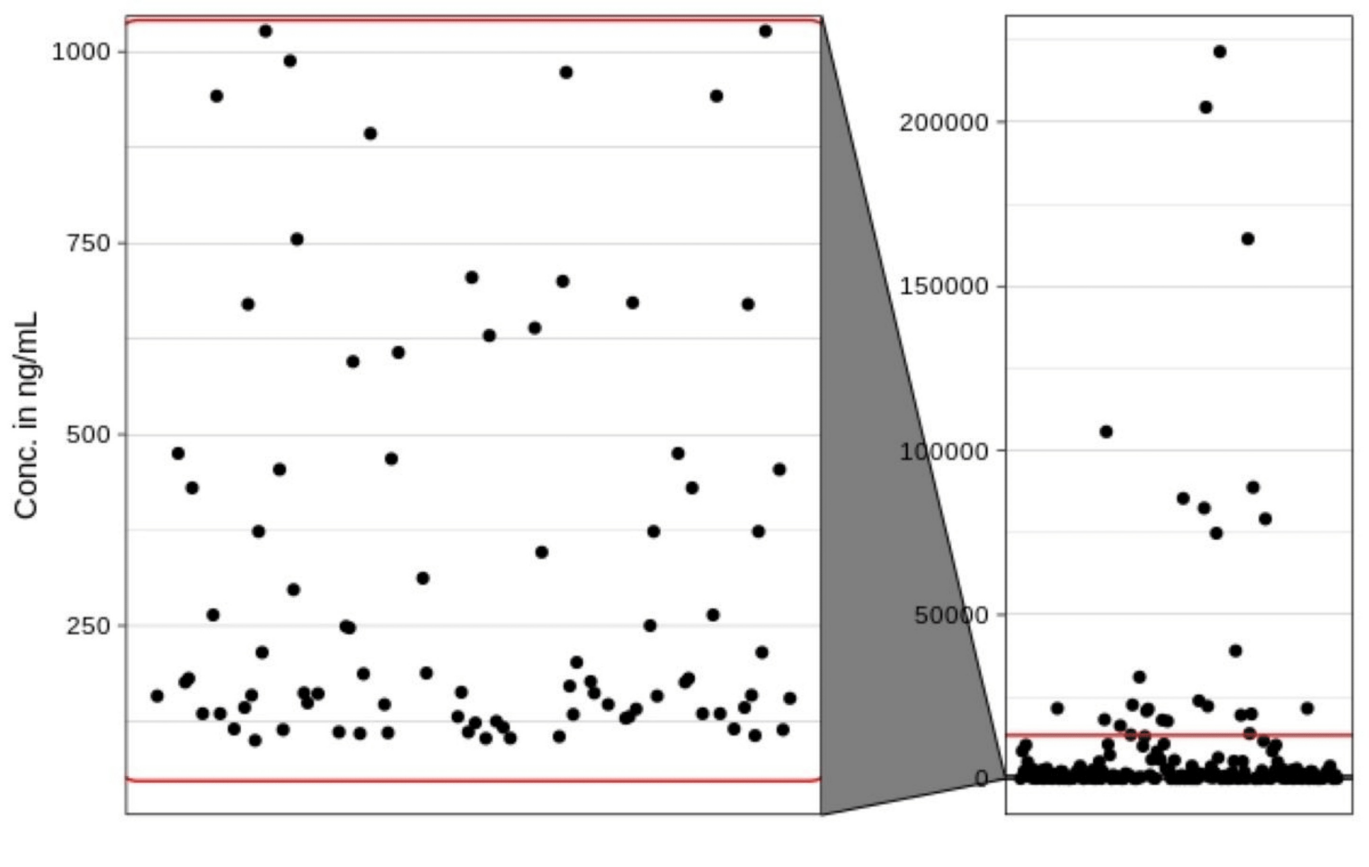
Individual values of the analyte detected with a zoom-in view of values in the range of linearity (indicated by the red line).

All positive samples were from male users (low to medium-income group, working or self-employed, and with a history of last use) in the 26-54-year age group. Because the distribution was highly skewed, we summarized the distribution using non-parametric statistics. The median concentration was 1,193.5 ng/mL with a lower quartile (first quartile) of 187.25 ng/mL and a third quartile of 5,177 ng/mL. We identified 18 values as outliers as they were higher than three times the third quartile. However, after collaborating with patient use patterns, we did not find these values as erroneous and matched with the abuse history, thus they have been retained in Figure 3.

## Discussion

GC-MS proved to be an effective technique for highly precise and quantitative measurements of tramadol in the urine sample. The accurate detection of tramadol in urine was made possible by the instrument’s high sensitivity, resolving power, and inherent accuracy. Investigating the peak interference from the endogenous urine substances helped confirm the method’s specificity. Figure 2 shows the representative chromatograms of tramadol in urine samples. Tramadol was successfully isolated using a bond-elute cartridge and SPE sample extraction (Figure 1).

Figures of merit

Under the selected optimum experimental conditions, an acceptable calibration curve was developed by spiking the urine sample with different concentrations of tramadol. Three similar extractions were performed for each concentration to attain a more precise calibration curve. In this step, the following figures of merit were evaluated. The calculated LOD based on the signal-to-noise ratio of 3 was obtained at 20 ng/mL. The linear range with the correlation coefficient (R2) ≥0.998 was 50-1,000 ng/mL (Figure 2). The relative recovery was 92.5% with a precision of 4.4%. The stability of tramadol samples under various storage conditions. The relative standard deviation (RSD) in low (30 ng/mL), medium (500 ng/mL), and high (750 ng/mL) concentration ranges (n = 3), with the results obtained being within the range of 5% (Table 1).

Clinical applicability

A total of 747 pre-screened patient samples (adulteration, multidrug, and tramadol) were utilized to test the analytical application’s performance. Results from the screening showed that tramadol abuse history ranged from 187.25 to 5,177.0 ng/mL, with a median of 1,193.5 ng/mL. Figure 3 illustrates the method’s capability of finding various ranges of values.

Rouini et al. [14,16] presented an LLE procedure for tramadol extraction in plasma with ethyl acetate and 1.0 M sodium hydroxide but reported poor recovery due to various reasons. Previous studies have described a GC-MS method for the determination of tramadol using solvent bar micro-extraction and reported a similar detection limit and RSD of 4.5% [9,17]. Further, previous studies have described the GC-MS method for tramadol analysis in human urine samples [11,12]. The samples were cleaned with diethyl ether, acid hydrolyzed with hydrochloric acid, and extracted with an isopropanol:dichloromethane ratio of 9:1. Tramadol recovery was 85.2% ± 5.4 (n = 3) and the detection limit was lowered to 12.5 pg, which is no longer required to detect tramadol abuse [18].

The determination of tramadol in human biological samples is important to obtain information on long-term abuse. Various methods have been reported for tramadol determination such as liquid chromatography-mass spectrometer [15], GC-MS [17,18], capillary electrophoresis [19], spectrophotometry, and electrochemistry [20]. However, it is necessary to develop a rapid, simple, and sensitive method for the determination of tramadol in biological samples. The presented method offered a better separation of tramadol without background disturbance. Compared to the previously published approaches, the present method had a faster run time and appropriate sensitivity. The specificity of the method was verified by investigating the peak interference from the endogenous substances.

Limitations

In this study, the other excreted metabolites (O-desmethyl tramadol) with better half-lives were not taken into consideration due to the unavailability of reference standards.

## Conclusions

The effectiveness of the SPE method in combination with GC-MS for analyzing tramadol in urine samples was assessed in this study. Compared to earlier extraction techniques, this method is thought to be a novel technique. The analytical results generated by this procedure featured high enrichment of the required analyte through SPE, discernible recovery, quick analysis times, simplicity, low matrix effect, efficient detection limit, order of magnitude, dynamic range, reproducibility, good sensitivity, and ease of use. We strongly recommend employing this approach in fields such as pharmacology labs and forensic medicine.
